# Anatomic relation between the external branch of the superior laryngeal nerve and the thyroid gland

**DOI:** 10.1590/S1808-86942011000200016

**Published:** 2015-10-19

**Authors:** Fabiana Estrela, Henrique Záquia Leão, Geraldo Pereira Jotz

**Affiliations:** 1 Master's degree in neuroscience, UFRGS. Clinical speech therapist; 2 Master's degree in otorhinolaryngological science, UNIFESP-EPM. Biologist and adjunct professor of human anatomy at ULBRA. Ex-professor of human anatomy at the Morphological Science Department, UFRGS; 3 Doctoral degree in otorhinolaryngology and head & neck surgery, UNIFESP-EPM. Post-doctoral studies in otorhinolaryngology, Pittsburgh University, US. Associate professor and head of the Morphological Science Department, UFRGS. Adjunct professor of otorhinolaryngology and head & neck surgery, ULBRA. Morphological Science Department (Departamento de Ciências Morfológicas), Rio Grande do Sul Federal University (UFRGS)

**Keywords:** cadaver, larynx, morphology, laryngeal nerves

## Abstract

**Aim:**

This prospective study investigated the anatomic relations between the external branch of the superior laryngeal nerve (EBSLN), the superior thyroid artery (STA) and the thyroid gland in human cadavers.

**Material and Methods:**

Twenty-two human cadavers aged over 18 years old, less than 24 hours after death.

**Results:**

The mean distance between the EBSLN and the superior pole of the thyroid gland was 7.68 ±3.07 mm. A tangent to the inferior edge of the thyroid cartilage between the EBSLN and the STA measured 4.24 ±2.67 mm. A line from the intersection of the EBSLN - related to the STA - to the superior pole of the thyroid gland measured 9.53 ±4.65 mm. A line from the EBSLN to the midline of the most caudal point of the thyroid cartilage measured 19.70 ±2.82 mm. A line from the RENLS to the midline on the most cranial point of the cricoid cartilage was 18.35 ±3.66 mm.

**Conclusion:**

There is a variable proximity relation between the EBSLN and the superior pole of the thyroid gland; this distance ranges from 3.25 to 15.75 mm. There was no evidence of significant variation between the measures in the ethnic groups comprising the sample.

## INTRODUCTION

The thyroid gland is closely related to vital structures of the neck, such as the cervical esophagus, the recurrent laryngeal nerve, the superior laryngeal nerve, major vessels and the parathyroid glands. For this reason, understanding the anatomy of these adjacent structures is paramount in thyroid gland surgery and other procedures in this region. Retrospective, prospective and clinical studies have investigated permanent and temporary palsy of the recurrent and superior laryngeal nerves as sequelae of thyroidectomies. The recurrent laryngeal nerve in particular has received much attention due to its immediate postoperative consequences. Damage to this nerve may cause temporary or permanent vocal fold paralysis and dysphonia.

Compared to the recurrent laryngeal nerve, the superior laryngeal nerve and its branches have not been given such attention in the surgical literature. This is possibly because detecting damage to the branches of the superior laryngeal nerve requires detailed investigation. Damage to the external branch of the superior laryngeal nerve (EBSLN) cause palsy of the cricothyroid muscle and alters lower airway protection mechanisms. This muscle tensions the vocal folds to produce high-frequency sounds; it also helps maintain the vocal folds under tension during phonation. Thus, EBSLN palsy may alter the ability to produce acute sounds and lead to dysphagia, especially with liquids.

Clader et al.^1^ found that 68 % of studied cases were at risk of surgical injury to the EBSLN, 12 % were at questionable risk, and 20 % were not at risk. Risk analysis was made based on the distance to the point of ligature of blood vessels, especially the superior thyroid artery (STA). The authors concluded that the EBSLN is frequently at risk when the STA is ligated, because of its frequent anatomical closeness to this artery at the usual ligature point during surgery of the thyroid. The authors, however, did not measure this distance.

Cernea^2^ carried out a study involving the dissection of 30 superior thyroid poles of 15 recent human cadavers to identify the EBSLN and analyze its anatomical relations with the superior pedicle of the thyroid gland. Based on an anatomical and surgical classification, one nerve (3 %) was not located, 18 (60 %) were type 1 (nerve crossing the superior thyroid vessels 1 cm above a horizontal plane along the border of the superior thyroid pole), 5 (17 %) were type 2a (nerve crossing the vessels less than 1 cm over the abovementioned plane), and 6 (20 %) were type 2b (nerve crossing the vessels below the abovementioned plane). This last anatomical configuration was considered as risky for iatrogenic injury during thyroidectomy.

Sun & Chang^3^ studied 60 cadavers and described a high rate of looping in the superior laryngeal nerve connecting with the lateral sympathetic chain in 98.3 % of cases. The loops were divided into three categories, in turn classified in 5 types and 17 subtypes according to the morphology. The authors added that care should be taken in these loops not to injure the nerve in thyroid gland surgery. Loops consisted of connections between the cervical sympathetic chain and the superior laryngeal nerve, located at a certain distance to the superior pole of the thyroid gland. The lateral portion of loops is posterior and lateral to the superior thyroid artery; the medial portion of the loops is anterior and medial to the artery. Loops give rise to the branch to the cricothyroid muscle and the gland branch close to the superior pole of the thyroid gland. The reported distance from the lower point of the loops of the EBSLN and the superior pole of the thyroid gland was about 1 cm.

Cernea et al.^4^ found that the probability of EBSLN injury is about 20 % depending on the distance between the nerve and the superior pole of the thyroid gland. These authors also stated that the nerve position bilaterally was symmetrical in 47 % of subjects and asymmetric in 53 % of subjects. These numbers suggest varying risk in each side in bilateral thyroidectomies.

McMinn et al.^5^ added that the superior laryngeal nerve is posterior to the superior thyroid artery, as the artery is close to the superior pole of the lateral lobe.

Williams et al.^6^ reported that the EBSLN, which is smaller than the internal branch, descends posterior to the sternothyroid muscle next to the superior thyroid artery, but at a deeper plane. This branch runs alongside the inferior pharyngeal constrictor muscle, perforating it and curving around the inferior thyroid tubercule to reach and innervate the cricothyroid muscle. The EBSLN also innervates the pharyngeal plexus and the inferior constrictor; it also connects with the superior cardiac nerve and the superior cervical sympathetic ganglion behind the common carotid artery.

Kokocharov et al.^7^ described the EBSLN as being very close to the superior thyroid artery and vein, usually in a medial position to these vessels. Inferiorly, it courses along the anterior surface of the inferior pharyngeal constrictor muscle or penetrates this muscle to reach the cricothyroid muscle. These authors have suggested that dissection of the blood vessels along the superior pole of the thyroid should be done at least for 1.5 to 2 cm from the entry point of these vessels into the thyroid capsule to facilitate exposure of the thyroid gland during thyroidectomies and to avoid injury to the EBSLN.

Thus, the purpose of this study of human cadavers was to investigate the anatomical relations between the EBSLN, the superior thyroid artery and the thyroid gland bilaterally, measuring the distances between these structures.

## MATERIAL AND METHODS

A descriptive cross-sectional observational study was carried out based on a sample consisting of 22 male human cadavers - 44 superior thyroid poles. Subjects were aged over 18 years, had died of extracervical causes, and were dissected within 24 hours of death.

Neck dissections were carried out bilaterally by the same researcher in all subjects to identify the EBSLN and its relation with the superior thyroid artery and the thyroid gland. To start the neck dissection, this area was washed with acetic acid before the incision; the cadaver was placed with the neck hyperextended. A longitudinal incision was made on the neck following the necropsy incision line; structures were opened in planes. The thyroid was identified and dissected to its superior pole, where the superior thyroid artery and the vagus nerve were identified next to the carotid artery and the internal jugular vein bilaterally; then the superior laryngeal nerve and its external branch was dissected until its insertion into the cricothyroid muscle.

Photographic documentation was made with Yashica^®^ Dental Eye II camera and colored Fuji^®^ ASA 400 film.

A non-digital 150 mm pachymeter was used to make five measurements in each side of the dissected area, as follows:

1 the shortest measurement from the EBSLN to the most cranial point of the thyroid gland lobe;
2 the shortest transverse measurement from the superior thyroid artery to the EBSLN along a tangent to the inferior border of the thyroid cartilage;
3 the shortest measurement from the crossing point between the superior thyroid artery and the EBSLN and the most cranial point of the thyroid lobe. If the superior thyroid artery and the EBSLN did not cross, the symbol “nc” (“no crossing”) was annotated in the data sheet;
4 the shortest measurement from the EBSLN to the midline of the neck at the most caudal point of the thyroid cartilage;
5 the shortest measurement from the EBSLN to the midline of the neck on the most cranial point of the cricoid cartilage.

The measurements were analyzed by comparing each according to the following variables: side (left or right), ethnic group (Caucasian or non-Caucasian) (Table 1), and age group (group 1 - 18 to 39 years; group 2 - 40 years or more) (Table 2). The age groups were set up because after the age 40 years the body - including the larynx - undergoes structural and physiological changes such as an increase in fat, a reduction in bone and muscle tissues, ossification of laryngeal cartilages, and other changes.

**Table 1:** Description of blocks according to the ethnic group:

Ethnic group	N
Caucasians	15
Non-Caucasians	7

Total	22

**Table 2:** Division of subjects into two age groups:

Age group	N
≤ 39 years	12
≥ 40 years	10

Total	22

Tables 1 and 2 (ethnic group and age), the body mass, the height, time elapsed since death, the cause of death, the abovementioned measurements, and identification of pictures were annotated and placed in the registry files at the Office of the Chief Medical Examiner in Porto Alegre. The researchers confirmed the body mass and height measurements.

The body mass index (BMI) was calculated as the relation between body mass and height and expressed as kg/m^2^.

All procedures were carried out by the researchers in the necropsy room of the Office of the Chief Medical Examiner in Porto Alegre.

### Ethical issues

The institutional review board of the Rio Grande do Sul Federal University (UFRGS) approved this study. The sample consisted entirely of subjects under the legal responsibility of the Office of the Chief Medical Examiner.

### Statistical analysis

The SAS System version 8.01 software and the SPSS for Windows version 8.0 software were used for the statistical analysis.

Pearson's correlation coefficient was applied to calculate the correlation among variables, as follows: the age, body mass, height, and BMI with measurements 1 to 5; and between sides for each measurement. Pearson's correlation coefficient ranges from -1 to 1: a negative sign indicates an inverse correlation (as one index increases the other decreases); a positive sign indicates a direct correlation (both indices increase). A value closer to 1 indicates a stronger correlation; a value close to 0 indicates a weaker correlation. The significance level for correlations was 5 %.

The t test for independent samples was applied to analyze relationships between groups 1 and 2 (by age) and other variables.

Analysis of variance (randomized block design at a 5 % significance level) was applied to verify the interaction among the variables “group” and “side”, and among the variables “ethnic group” and “side” for the five measurements.

## RESULTS

The causes of death in the sample were: multiple organ failure and retroperitoneal malignancy, death from firearm with bullets in the head and abdomen, chronic liver cirrhosis, intracranial hemorrhage, intracranial hemorrhage due to firearm injury, sudden death, heart failure. The age in the full sample, expressed as the mean ± standard deviation, was 39 ± 18.91 years. Table 3 shows other data characterizing the sample: time elapsed after death (min.), body mass (kg), height (m), and the BMI (kg/m^2^), expressed as the mean ± standard deviation.

**Table 3:** Characterization of the sample in time elapsed after death, body mass, height, and the BMI. The t test for independent samples was applied to analyze the relationships between groups 1 and 2 according to the age group and the variables mentioned above:

Side	Age groups				Total
	(1) ≤ 39 years		(2) ≥ 40 years		
	Mean	Standard deviation	Mean	Standard deviation	^*p*^
Time	472.50	268.67	461.00	270.44	0.922
Mass	68.17	6.40	58.51	14.60	0.076
Height	1.75	0.05	1.70	0.06	0.035*
BMI	22.26	1.93	20.15	3.97	0.148

* the minimum significance level in the t test was *p* < 0.05

The t test (*p*=0.035) showed that subjects in the ≤ 39-year group were significantly higher than subjects in the ≥ 40-year group (Table 3).

There was a significant, direct, and mean correlation between height and measurement 4 (from the EBSLN to the midline of the neck along the most caudal point of the thyroid cartilage) in the right, irrespective of age - as height increases, so does measurement 4 on the right.

There was an inverse correlation between the body mass and BMI with measurement 1 (shortest measurement from the EBSLN to the most cranial point of the thyroid gland lobe) in the left, irrespective of age - as the body mass and BMI increase, measurement 1 decreases in the left.

The BMI itself did not correlate significantly with any measurement.

There was a significant direct, and mean correlation between age and measurement 2 (shortest transverse measurement from the superior thyroid artery to the EBSLN along a tangent to the inferior border of the thyroid cartilage) in the right - as age increased, so did measurement 2 in the right.

There was a significant direct, and mean correlation between height and measurement 4 (from the EBSLN to the midline of the neck at the most caudal point of the thyroid cartilage) in the right, irrespective of age - as height increased, so did measurement 4 in the right.

There was a significant direct, and mean correlation between measurement 5 (from the EBSLN and the midline of the neck on the most cranial point of the cricoid cartilage) in the right and the same measurement contralaterally, irrespective of age - as measurement 5 in the right increased, so did measurement 5 in the left.

Analysis of variance (randomized block design at a 5 % significance level) revealed no significant interactions between ethnic groups and side for all measurements. The main effects as applied to ethnic groups and side were also not significant - there were no significant differences in measurements irrespective of the side or the ethnic group. This suggests that there are no significant measurement variations among ethnic groups or between sides in the same subjects.

**Figure 2:**
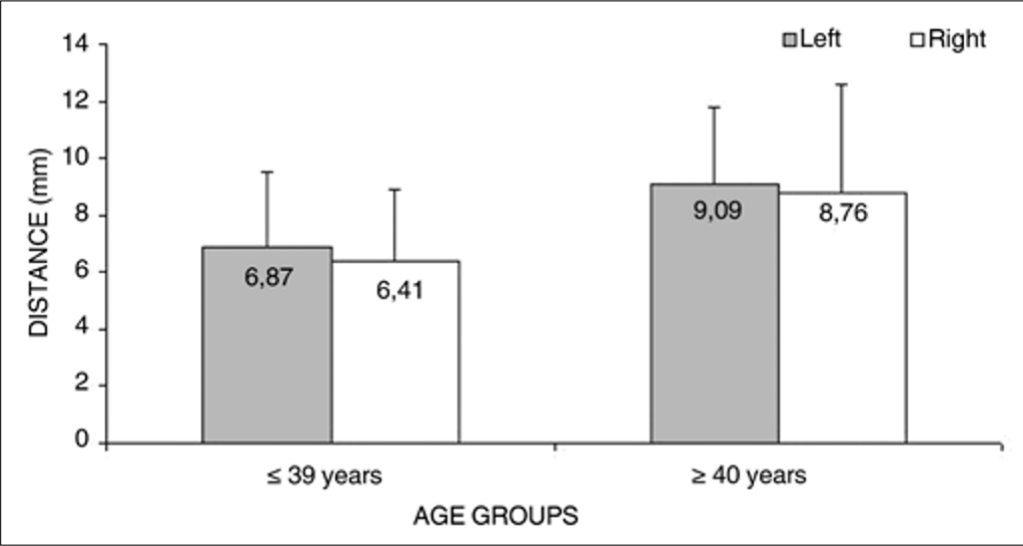
Measurement 1 relative to age and side, in mm (mean ± standard deviation) - measurement 1 (the shortest measurement from the EBSLN to the most cranial point of the thyroid gland lobe).

Analysis of variance (randomized block design at a 5 % significance level) showed no significant interaction between the variables age group and side for measurement 1 (shortest measurement from the EBSLN to the most cranial point of the thyroid gland lobe). In the main effects, only age was significant - irrespective of the side, the ≤ 39-year group had a significantly lower mean (6.64 + 2.54) than the ≥ 40-year group (8.92 + 3.24) (Table 4 and Fig. 2). The shortest mean distance from the EBSLN and the most cranial point of the thyroid gland lobe (measurement 1) was 7.68 + 3.07 mm (mean + standard deviation).

**Table 4:** Measurement 1 relative to age and side, in mm, expressed as the mean ± standard deviation:

Side	Age groups				Total	
	(1) ≤ 39 years		(2) ≥ 40 years			
	Mean	Standard deviation	Mean	Standard deviation	Mean	Standard deviation
Left	6.87	2.67	9.09	2.69	7.88	2.85
Right	6.41	2.50	8.76	3.85	7.48	3.32

Total	6.64*	2.54	8.92	3.24	7.68	3.07

**Figure 1:**
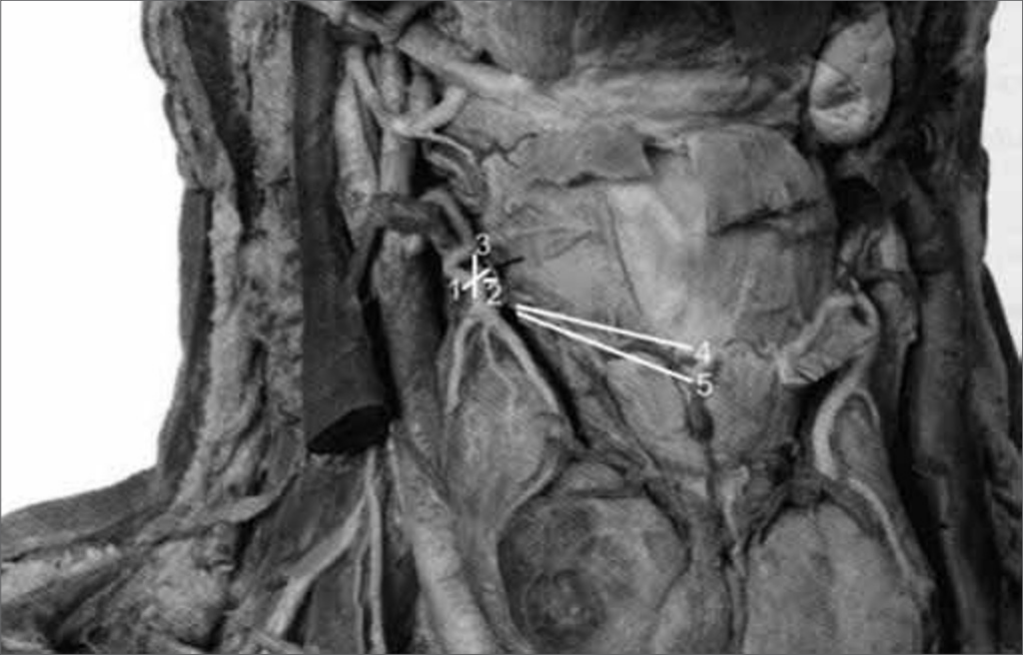
Antero-lateral area of the dissected neck, exposing the thyroid gland and associated structures. White lines and numbers correspond to measurements 1 to 5 described above. - (Adapted from McMinn, Hutchings & Logan, 1994)

**Figure 3:**
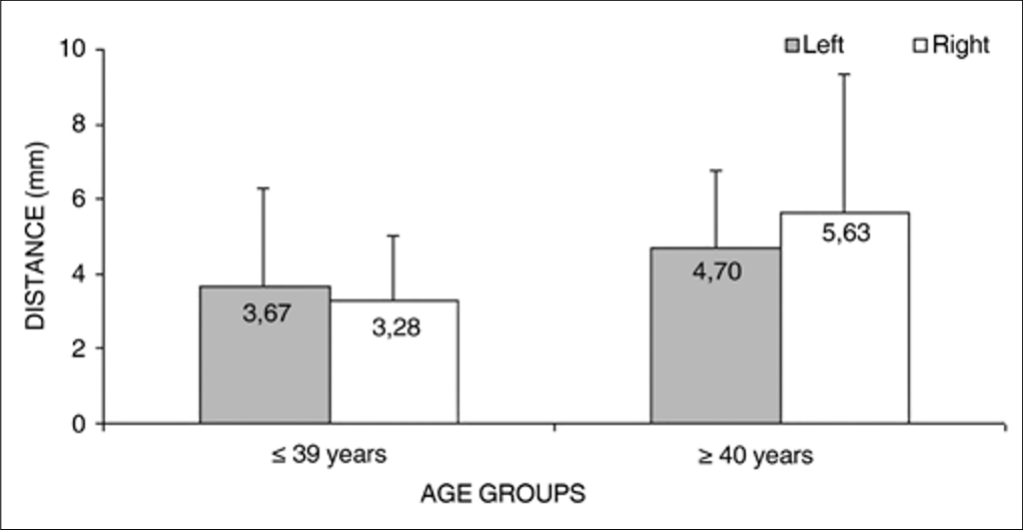
Measurement 2 relative to age and side, in mm (mean ± standard deviation) - measurement 2 (the shortest transverse measurement from the superior thyroid artery to the EBSLN along a tangent to the inferior border of the thyroid cartilage).

Analysis of variance (randomized block design at a 5 % significance level) showed no significant interaction between the variables age group and side for measurement 2 (shortest transverse measurement from the superior thyroid artery to the EBSLN along a tangent to the inferior border of the thyroid cartilage). In the main effects, the variable age group tended strongly towards a lower mean measurement 2 (3.47 + 2.18) in the ≤ 39-year group compared to the ≥ 40-year group (5.16 + 2.95) (Table 5 and Fig. 3). The mean value of the shortest transverse measurement from the superior thyroid artery to the EBSLN along a tangent to the inferior border of the thyroid cartilage (measurement 2) was 4.24 + 2.67 mm (mean + standard deviation).

**Table 5:** Measurement 2 relative to age group and side. in mm. expressed as the mean ± standard deviation:

Side	Age groups				Total	
	(1) ≤ 39 years		(2) ≥ 40 years			
	Mean	Standard deviation	Mean	Standard deviation	Mean	Standard deviation
Left	3.67	2.61	4.70	2.06	4.14	2.38
Right	3.28	1.74	5.63	3.70	4.34	2.98

Total	3.47	2.18	5.16	2.95	4.24	2.67

**Figure 4:**
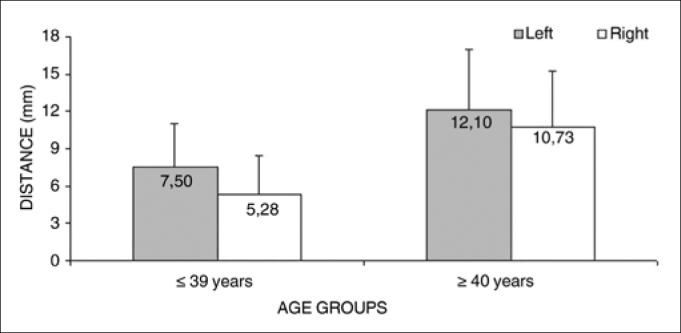
Measurement 3 relative to age and side, in mm (mean ± standard deviation) - measurement 3 (the shortest measurement from the crossing point between the superior thyroid artery and the EBSLN and the most cranial point of the thyroid lobe).

Measurement 3 (shortest measurement from the crossing point between the superior thyroid artery and the EBSLN and the most cranial point of the thyroid lobe) was studied only subjects where the superior thyroid artery crossed the EBSLN, as follows: six subjects in the group with subjects aged from 18 to 39 years, and nine subjects in the group with subjects aged from 40 to 89 years, totaling 15 subjects. Analysis of variance (randomized block design at a 5 % significance level) showed no significant interaction between the variables age group and side. In the main effects, only age group was significant; thus, irrespective of the side, the group aged ≤ 39 years had a significantly lower mean (6.76 + 3.40) compared to the group aged from 40 to 89 years (11.32 + 4.56) (Table 6 and Fig. 4).

**Table 6:** Medida 3 quanto à faixa etária e lado. em mm. expressa em média±desvio-padrão.

Side	Age groups				Total	
	(1) ≤ 39 years		(2) ≥ 40 years			
	Mean	Standard deviation	Mean	Standard deviation	Mean	Standard deviation
Left	7.50	3.53	12.10	4.89	9.80	4.72
Right	5.28	3.19	10.73	4.54	9.25	4.79

Total	6.76	3.40	11.32	4.56	9.53	4.65

The mean value of the shortest measurement from the crossing point between the superior thyroid artery and the EBSLN and the most cranial point of the thyroid lobe (measurement 3) was 9.53 + 4.65 mm (mean + standard deviation).

**Figure 5:**
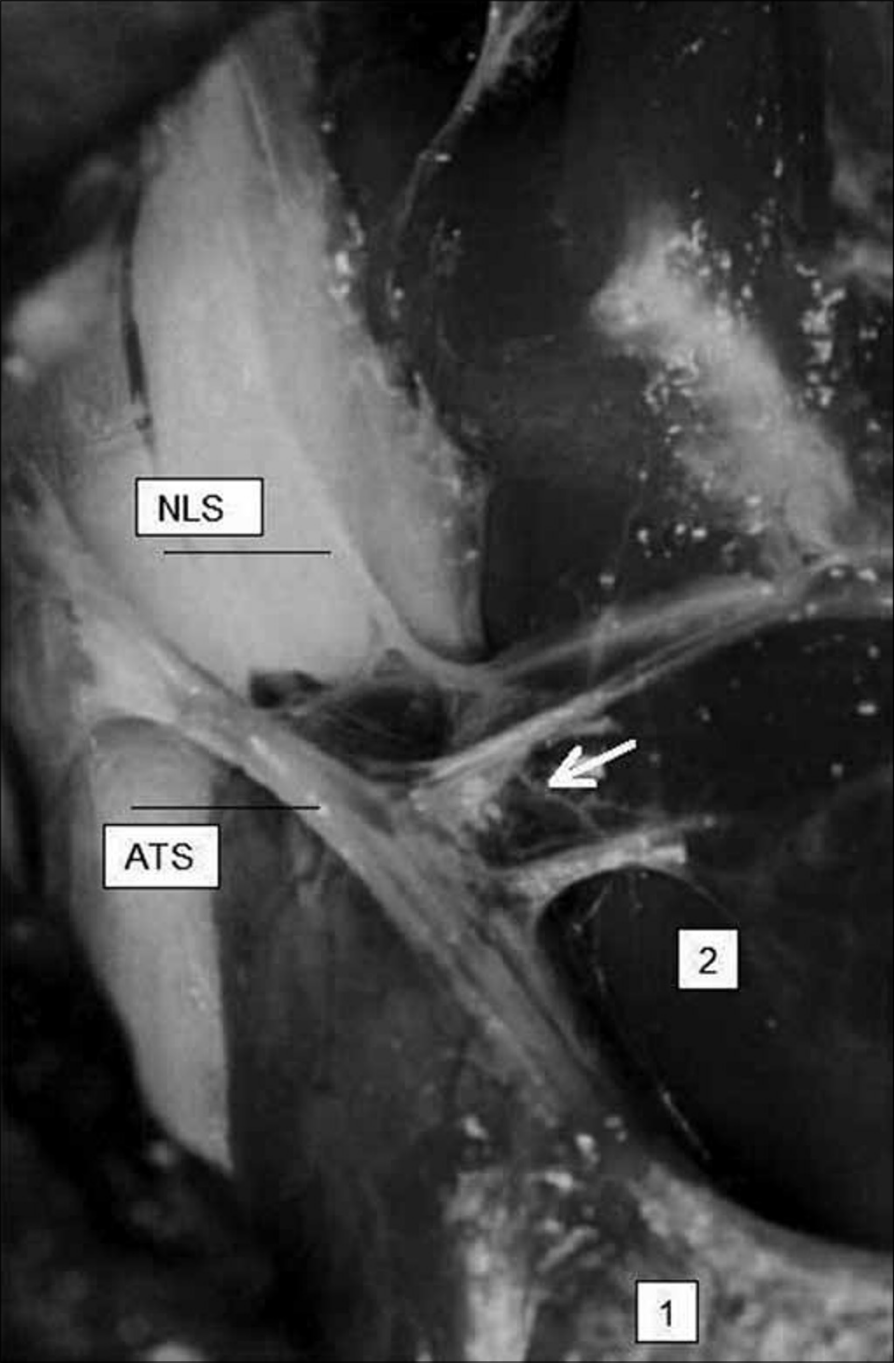
Right-lateral view of the neck at the larynx, showing the parallel and posterior trajectory of the external laryngeal nerve (white arrow) relative to the superior thyroid artery. Key: (1) tip of the right superior pole of the thyroid gland; (2) right cricothyroid muscle; SLN, superior laryngeal nerve.

**Figure 6:**
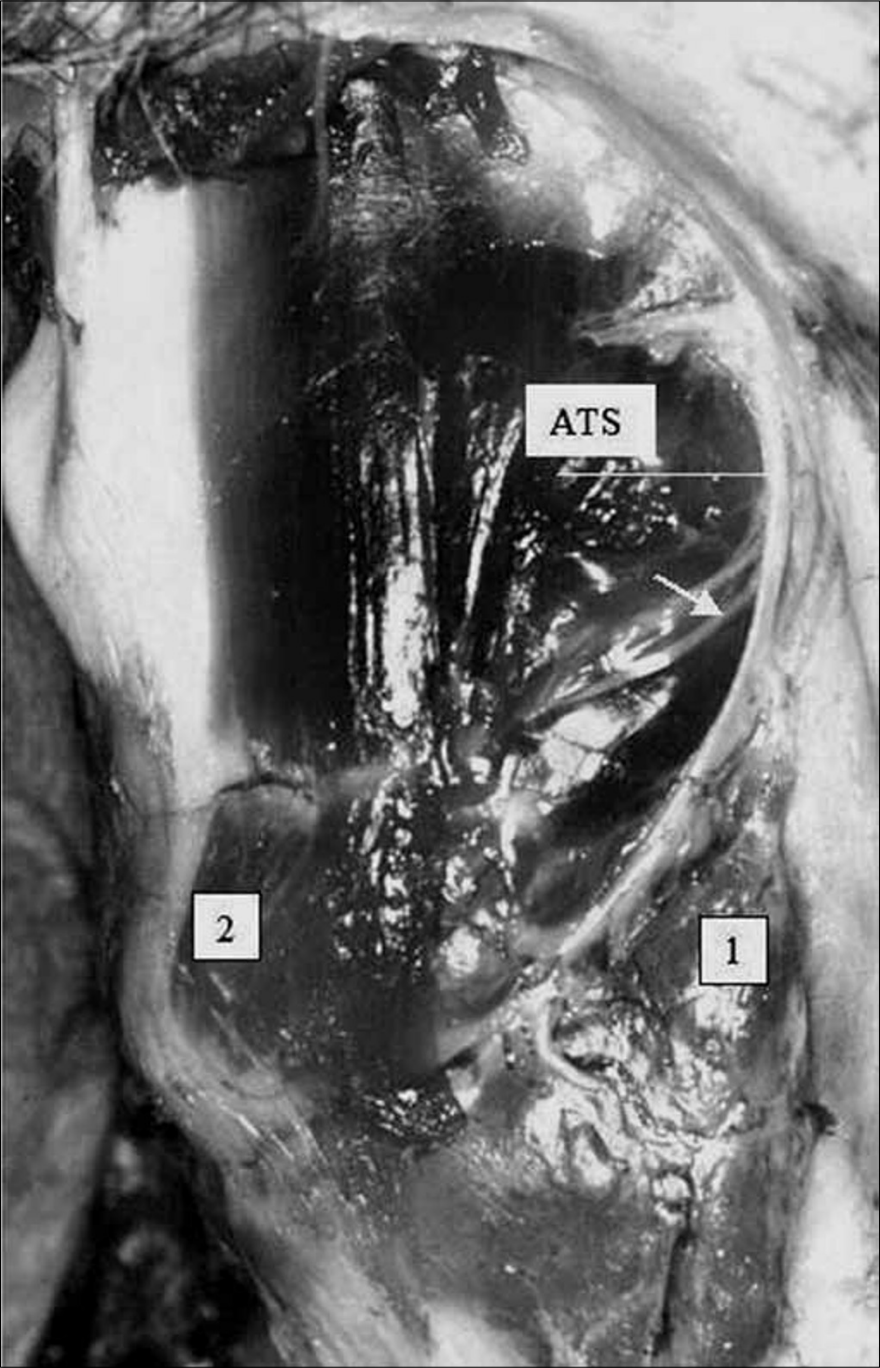
Left lateral view of the neck at the larynx, showing the external laryngeal nerve (white arrow) crossing the superior thyroid artery. Key: (1) tip of the left superior pole of the thyroid gland; (2) left cricothyroid muscle; SLN, superior laryngeal nerve.

We noted that after branching from the superior laryngeal nerve, the external branch runs posteriorly to the internal and external carotid arteries and courses a parallel path, posterior and very close to the superior thyroid artery (Fig. 5). The EBSLN may cross the superior thyroid artery posteriorly close to the superior pole of the thyroid gland, as seen in measurement 3 (Fig. 6). The relation between the EBSLN and the superior thyroid artery was that the EBSLN crossed the superior thyroid artery posteriorly and deeply on the left side in 54.54 % (12) of subjects; this also occurred in 50 % (11) of subjects on the right side.

The EBSLN crossed the superior thyroid artery on both sides in 36.36 % (8 subjects) of 22 subjects; unilateral crossing was seen in 31.81 % (7 subjects), and no crossing was observed in 31.81 % (7 subjects). Thus, crossing of the superior thyroid artery and the EBSLN may be present bilaterally or unilaterally or may be absent, in equal proportion. If absent, the EBSLN courses parallel, posterior and deep to the superior thyroid artery. Thus, the anatomical relation between the EBSLN and the superior thyroid artery close to the superior pole of the thyroid gland should be taken carefully into account during surgery of this area because of such variability.

**Figure 7:**
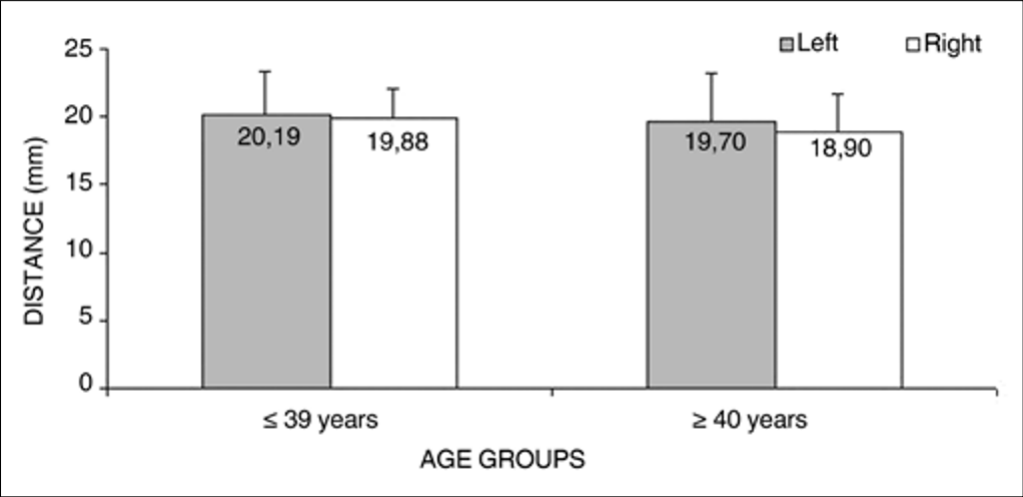
Measurement 4 relative to age and side, in mm (mean ± standard deviation) - measurement 4 (the shortest measurement from the EBSLN to the midline of the neck at the most caudal point of the thyroid cartilage).

Analysis of variance (randomized block design at a 5 % significance level) showed no significant interaction between the variables age group and side in measurement 4 (shortest measurement from the EBSLN to the midline of the neck at the most caudal point of the thyroid cartilage). The main effects were also not significant (Table 7 and Fig. 7).

**Table 7:** Measurement 4 relative to age group and side. in mm. expressed as the mean ± standard deviation:

Side	Age groups				Total	
	(1) ≤ 39 years		(2) ≥ 40 years			
	Mean	Standard deviation	Mean	Standard deviation	Mean	Standard deviation
Left	20.19	3.09	19.70	3.46	19.97	3.20
Right	19.88	2.14	18.90	2.74	19.43	2.42

Total	20.04	2.61	19.30	3.07	19.70	2.82

The mean measurement from the EBSLN to the midline of the neck at the most caudal point of the thyroid cartilage (measurement 4) was 19.70 + 2.82 mm (mean + standard deviation).

**Figure 8:**
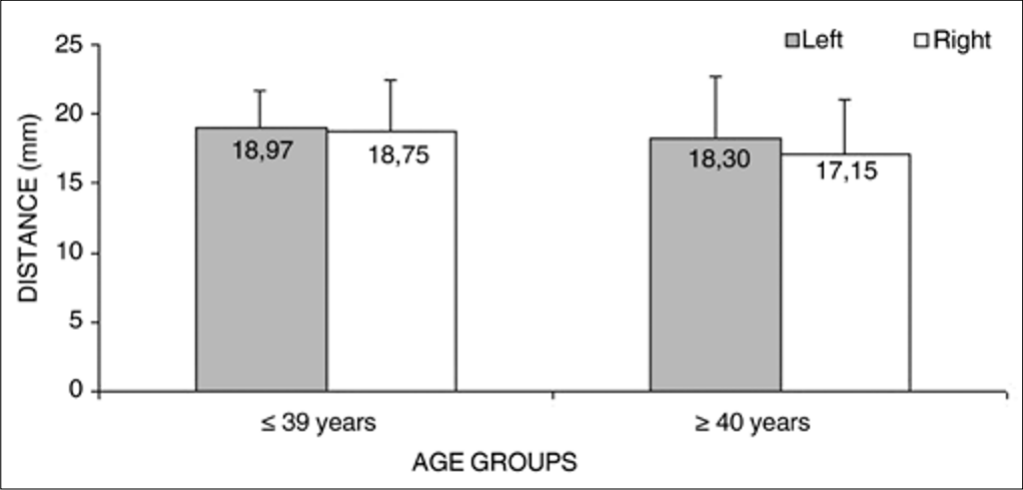
Measurement 5 relative to age and side, in mm (mean ± standard deviation) - measurement 5 (the shortest measurement from the EBSLN to the midline of the neck on the most cranial point of the cricoid cartilage).

Analysis of variance (randomized block design at a 5 % significance level) showed no significant interaction between the variables age group and side in measurement 5 (shortest measurement from the EBSLN to the midline of the neck on the most cranial point of the cricoid cartilage). The main effects were also not significant (Table 8 and Fig. 8).

**Table 8:** Measurement 5 relative to age group and side, in mm, expressed as the mean ± standard deviation:

Side	Age groups				Total	
	(1) ≤ 39 years		(2) ≥ 40 years			
	Mean	Standard deviation	Mean	Standard deviation	Mean	Standard deviation
Left	18.97	2.76	18.30	4.45	18.67	3.55
Right	18.75	3.73	17.15	3.93	18.03	3.82

Total	18.86	3.21	17.73	4.13	18.35	3.66

The shortest measurement from the EBSLN to the midline of the neck on the most cranial point of the cricoid cartilage (measurement 5) was 18.35 + 3.66 mm (mean + standard deviation).

## DISCUSSION

Most authors until the 1980s did not describe the EBSLN and its relation with the superior thyroid artery and the thyroid gland in any detail.^1^, ^8^, ^9^, ^10^, ^11^ They merely reported its descending path from its origin in the superior laryngeal nerve to its insertion in the cricothyroid muscle passing through the inferior pharyngeal constrictor muscle and the pharyngeal plexus. In our study we underlined the important close relation between the EBSLN and the superior thyroid artery, as well as its variable crossing posterior and deep to the superior thyroid artery.

The EBSLN crossed the superior thyroid artery on both sides in 36.36 % (8) of subjects; it crossed the artery unilaterally in 31.81 % (7) patients, and it did not cross the artery on both sides in 31.81 % (7) of subjects. Thus, the EBSLN may cross the superior thyroid artery bilaterally, unilaterally, or not at all in equal percentages; in the latter situation, the EBSLN is parallel, posterior, and deep to the superior thyroid artery. Cernea^2^ reported that the EBSLN had a similar path on both sides and crossed the thyroid vessels superiorly in all cases in only 7 (47 %) of 15 cadavers in their study, which differed from out results. Sun & Chang^3^, based on a study of 60 cadavers (120 sides), also reported that the position of the EBSLN varied, and that the superior laryngeal nerve had a high rate of looping in its connections to the lateral sympathetic chain; the lateral portion of these loops were posterior and lateral to the superior thyroid artery, similar to our findings.

The EBSLN was found on both sides in all 22 subjects of our sample; it was always in a close relation with the superior thyroid artery (posterior and deep to it), running parallel or crossing it in equal measure. Bellantone et al.^12^ investigated the incidence of injury to the EBSLN by using two different surgical approaches; the EBSLN was not clearly identified in 11.6 % of cases. McMinn et al.^5^ pointed out - as in our study - that the superior laryngeal nerve was posterior to the superior thyroid artery, especially at the point where it is closest to the superior pole of the lateral thyroid lobe. Williams et al.^6^ showed that the EBSLN courses posterior to the superior thyroid artery at a deeper plane, as we also reported. Monfared et al.^13^ also showed that the EBSLN coursed deep and parallel to the superior thyroid artery. Kokocharov et al.^7^ described the EBSLN close to the superior thyroid artery and vein - usually medial to these vessels. Poyraz & Çalguner^14^ also reported that the EBSLN was medial to the superior thyroid artery in 71.9 % of subjects, and that it coursed between the branches of the superior thyroid artery in 28.1 % of cases.

The shortest measurement from the EBSLN to the most cranial point of the thyroid gland lobe (measurement 1) was 7.68 + 3.07 mm (mean + standard deviation); the ≤ 39 year group had a significant lower mean than the group aged 40 to 89 years. Sun & Chang^3^ reported that the distance from the lowest point of EBSLN looping and the superior pole of the thyroid gland was on average 1 cm - 3 mm more than we found. Cernea et al. (1992) noted that the injury rate of the EBSLN is about 20 %, based on the distance between the nerve and the superior pole of the thyroid gland; these authors, however, did not report data for this measurement. Kokocharov et al.^7^ suggested that the distance from the EBSLN, next to the superior thyroid artery and veins, and the thyroid gland capsule was 1.5 to 2 cm, almost double the measurement we found. Monfared et al.^13^ stated that the most vulnerable injury site for the EBSLN during thyroid surgery was a point close to the superior pole of the thyroid, because the mean distance from this structure to the EBSLN was less than 10 mm. After originating from the superior laryngeal nerve, the EBSLN runs posterior to the internal and external carotid arteries, and courses parallel and close to the superior thyroid artery. We also found that the EBSLN runs parallel, deep and posterior to the superior thyroid artery; the EBSLN may, however, run parallel or cross this artery, and variations may be present between the right and left side of the same subject. Furlan, Cordeiro & Brandão^15^ have stated that this measurement may be shorter when the thyroid gland is larger - in this case, the EBSLN tends to be closer to the thyroid.

The mean value of the shortest transverse measurement from the superior thyroid artery to the EBSLN along a tangent to the inferior border of the thyroid cartilage (measurement 2) was 4.24 + 2.67 mm (mean + standard deviation); the mean value of the shortest measurement from the crossing point between the superior thyroid artery and the EBSLN and the most cranial point of the thyroid lobe (measurement 3) was 9.53 + 4.65mm (mean + standard deviation). A few authors have reported that the EBSLN and the superior thyroid artery are closely related, which should be noted during surgery of the thyroid gland to avoid damaging the superior laryngeal nerve. These authors did not, however, report measurement data between these structures or other measureme nts.^1^’^2^’^3^’^6^’^7^’^10^’^11^’^13^’^14^’^16^’^17^ We noted in our study how close the EBSLN and the superior thyroid artery are by providing a more objective measurement.

The results showed that the group aged 39 years or less tended to have a lower mean (3.47 + 2.18 mm) compared to the group aged 40 to 89 years (5.16 + 2.95 mm). A significant, direct, and mean correlation was found between age and measurement 2 on the right side; with age, measurement 2 on the right side appears to increase. A possible explanation for this increased distance between the superior thyroid artery and the EBSLN as age progresses is that body fat between the superior thyroid artery and the EBSLN close to adjacent muscle may increase. According to Wilmore & Costill,^18^ body fat increases with age because of extra intake in the diet, decreased physical activity, and lower ability of the body to mobilize fat reserves; the amount of bone and muscle also decrease with age.

Analysis of variance of measurement 3 (the shortest measurement from the crossing point between the superior thyroid artery and the EBSLN and the most cranial point of the thyroid lobe) indicated that the group aged 39 years or less had a significantly lower mean measurement (6.76 + 3.40 mm) compared to the group aged from 40 to 89 years (11.32 + 4.56 mm), irrespective of the side. The sample size for this measurement does not allow us to state that there is any morphological change in these anatomical structures after age 40 years. A hypothesis is the status of adjacent fat and muscles, any change of which could result in a reduction of the distance from the crossing point between the superior thyroid artery and the EBSLN and the most cranial point of the thyroid lobe. As noted above, Wilmore & Costill^18^ have stated that body fat increases with age because of extra intake in the diet, decreased physical activity, and lower ability of the body to mobilize fat reserves.

There was an inverse correlation between body mass and the BMI with measurement 1 (the shortest measurement from the EBSLN to the most cranial point of the thyroid gland lobe) on the left side, irrespective of age; as body mass and the BMI increased, measurement 1 on the left side decreased. Such relationship where the distance between the EBSLN and the thyroid gland is decreased and the body mass and BMI are increased may be explained by a possible presence of more fat tissue in adjacent muscles, which alters the position of the EBSLN, displacing it closer to the thyroid gland. The BMI, however, did not correlate significantly with any other measurement.

There was a significant, direct and mean correlation between height and measurement 4 (the shortest measurement from the EBSLN to the midline of the neck at the most caudal point of the thyroid cartilage) o the right side, irrespective of age; as height increased, the shortest measurement from the EBSLN to the midline of the neck at the most caudal point of the thyroid cartilage on the right side also increased. Furlan, Cordeiro & Brandão (2003) found a statistically significant difference in height when related with intrinsic risk factors for injury to the EBSLN during thyroidectomy; these authors noted that the EBSLN is further from the superior pole of the thyroid gland in higher subjects. Thus, the EBSLN should be further from the most caudal point of the thyroid cartilage, as seen in our study. According to Gardner, Gray & O’Rahilly,^19^ the medial surface of the lobes of the thyroid gland are related directly with the larynx, specifically with the laminae of the thyroid cartilage and the cricoid cartilage.

It could be suggested that measurement 4 would be larger in group 1 (lower age and increased height) on the right side, since this measurement increased with height. This difference was not seen in relation to the BMI - a ratio between body mass and height of subjects. Analysis of variance (randomized block design at a 5 % significance level) showed that there was no significant interaction between the variables group and side relative to measurement 4; thus, although subjects in group 1 were significantly higher, and measurement 4 on the right side correlated directly with height, analysis of variance showed no significant difference between groups 1 and 2 - in our sample measurement 4 did not change as age advanced.

There was a significant, direct and mean correlation between measurement 5 (the shortest measurement from the EBSLN to the midline of the neck on the most cranial point of the cricoid cartilage) on the right side and the left side, irrespective or age; in other words, measurement 5 was proportional between both sides in the same subject. This seems important for surgical planning, because when checking the location of the EBSLN relative to the cricoid cartilage and the midline of the neck on one side, a surgeon may find the contralateral EBSLN more easily.

Analysis of variance of five measurements relative to the variable ethnic group showed that there were no statistically significant differences in measurements of Caucasians and non-Caucasians. This finding suggests that there are no significant measurement variations in different ethnic groups. Thus, comments about the anatomical measurements in this study may be generalized to the ethnic groups that were included in the study.

Furlan, Cordeiro & Brandão,^15^ based on the anatomical relationship between the EBSLN and the thyroid gland, found no statistically significant difference among ethnic groups in a study on intrinsic risk factors for surgical injury to this nerve.

Our data revealed differences among and within subjects with regards to the path of the EBSLN; these differences may be related with the high rate of intraoperative injury during procedures close to this nerve. We found concerns in the literature about the importance of detailed anatomical mapping as a basis for surgical planning of the thyroid gland and cervical structures close to the EBSLN, the superior thyroid artery, and the superior pole of the thyroid gland.^2^’^4^’^7^, ^12^, ^13^, ^15^, ^16^, ^17^, ^20^ Cernea^2^ also added that the only effective method for avoiding iatrogenic injury to the EBSLN during thyroidectomy is to systematically and objectively search for this nerve around the superior pole of the thyroid gland by using a neurostimulator.

It is hoped that this study of the anatomical relationships between the EBSLN, the superior thyroid artery, and the thyroid gland, in adult human cadavers, may contribute to surgical planning, with the aim of reducing injury to this nerve during surgery of the thyroid gland or adjacent structures.

## CONCLUSION

The distance between the EBSLN and the superior pole of the thyroid gland ranged from 3.25 to 15.75mm.

The EBSLN may cross the superior thyroid artery on both sides, on one side only, or not at all, in equal proportion; in the latter, the EBSLN courses parallel and deep to the superior thyroid artery.

Irrespective of the side, the mean value of measurement 1 (the shortest measurement from the EBSLN to the most cranial point of the thyroid gland lobe) was significantly lower in the group aged from 18 to 39 years compared to the group aged from 40 to 89 years.

The mean value of measurement 2 (the shortest transverse measurement from the superior thyroid artery to the EBSLN along a tangent to the inferior border of the thyroid cartilage) tended to be lower in the group aged from 18 to 39 years compared to the group aged from 40 to 89 years.

Measurement 2 on the right correlated directly and significantly with age: it tended to increase as age advanced.

The mean value of measurement 3 (the shortest measurement from the crossing point between the superior thyroid artery and the EBSLN and the most cranial point of the thyroid lobe) was significantly lower in the group aged from 18 to 39 years compared to the group aged from 40 to 89 years, irrespective of the side.

Measurement 4 (the shortest measurement from the EBSLN to the midline of the neck at the most caudal point of the thyroid cartilage) on the right correlated significantly and directly with height - as height increases the measurement from the EBSLN to the midline of the neck at the most caudal point of the thyroid cartilage also increases.

Measurements 4 (the shortest measurement from the EBSLN to the midline of the neck at the most caudal point of the thyroid cartilage) and 5 (the shortest measurement from the EBSLN to the midline of the neck on the most cranial point of the cricoid cartilage) showed no significant interaction with the variables age or side, except for measurement 5 in that it varied directly and proportionally with the sides within the same subject.

There were no significant measurement variations among ethnic groups in the sample, and between sides within the same subjects.

There was an inverse correlation between the BMI and measurement 1 on the left side, irrespective of age; as the BMI increases, measurement 1 on the left decreases.

The BMI did not correlate significantly with any of the other measurements.
